# Circulating microRNA Profiles as Diagnostic Tools for High-Grade Cervical Lesions and HPV Genotype Stratification

**DOI:** 10.3390/cells15090849

**Published:** 2026-05-06

**Authors:** Annika Tamenang, Vanessa Vohl, Charlotte Schwartz, Jolanthe Kropidlowski, Anna Jaeger, Katharina Hintelmann, Eik Vettorazzi, Yvonne Goy, Cordula Petersen, Sven Peine, Klaus Pantel, Barbara Schmalfeldt, Linn Woelber, Harriet Wikman, Katharina Effenberger

**Affiliations:** 1 Department of Tumor Biology, University Medical Center Hamburg-Eppendorf, Martinistr. 52, 20246 Hamburg, Germany; annika.tamenang@stud.uke.uni-hamburg.de (A.T.); vanessa.vohl@stud.uke.uni-hamburg.de (V.V.); charlotte.schwartz@stud.uke.uni-hamburg.de (C.S.); j.kropidlowski@uke.de (J.K.); pantel@uke.de (K.P.); h.wikman@uke.de (H.W.); 2 Department of Gynecology, University Medical Center Hamburg-Eppendorf, Martinistr. 52, 20246 Hamburg, Germany; a.jaeger@uke.de (A.J.); b.schmalfeldt@uke.de (B.S.); l.woelber@uke.de (L.W.); 3 Department of Radiotherapy and Radiation Oncology, University Medical Center Hamburg-Eppendorf, Martinistr. 52, 20246 Hamburg, Germany; katharina.hintelmann@uke.de (K.H.); yvonne.goy@gmx.de (Y.G.); cordula.petersen@uke.de (C.P.); 4 Department of Medical Biometry and Epidemiology, University Medical Center Hamburg-Eppendorf, Martinistr. 52, 20246 Hamburg, Germany; e.vettorazzi@uke.de; 5 Institute of Transfusion Medicine, University Medical Center Hamburg-Eppendorf, Martinistr. 52, 20246 Hamburg, Germany; s.peine@uke.de; 6 Dysplasie Center at the Jerusalem Hospital, Moorkamp 2-6, 20357 Hamburg, Germany

**Keywords:** cervical cancer, HSIL, precancerous cervical lesions, CIN, high-risk HPV, liquid biopsy, microRNA, circulating microRNA, molecular biomarkers, miRNA panels

## Abstract

**Highlights:**

**What are the main findings?**

- A three-miRNA panel (miR-21, miR-205, and miR-218) improves the detection of HSIL and cervical cancer compared to individual biomarkers.
- Circulating miRNA expression patterns are associated with HPV status, including HPV16/18 positivity and multiple HPV infections.

**What are the implications of the main findings?**

- Circulating miRNA signatures represent a promising minimally invasive approach to refine risk stratification in cervical dysplasia.
- Integration of miRNA biomarkers with HPV-based screening may enhance early detection and clinical surveillance strategies.

**Abstract:**

Persistent high-risk human papillomavirus (hr-HPV) infection drives cervical carcinogenesis, yet improved molecular biomarkers are needed to define high-risk groups. Circulating microRNAs (miRNAs), stable in blood and involved in carcinogenic pathways, represent promising liquid biopsy biomarkers. This study assessed five miRNAs for distinguishing high-grade squamous cell intraepithelial lesions (HSILs) and cervical cancer from healthy controls and for HPV stratification. Circulating miRNAs were quantified in blood samples from 80 women (38 HSIL, 10 cervical cancer, and 32 controls). Relative expression by disease and HPV status was measured by RT-qPCR and normalized to miRNA-23a. Diagnostic performance of single and combined miRNAs was evaluated by logistic regression and ROC curve analysis. Three circulating miRNAs (miR-21, miR-205, and miR-218) were found to be significantly differentially dysregulated in the patient cohorts. A combination of the three markers showed the best diagnostic value for HSIL (AUC of 0.81, sensitivity of 79%, and specificity of 71%) and cancer (AUC of 0.81, sensitivity of 90%, and specificity of 65%). Whereas miR-205 was significantly associated with HPV16/18 in HSIL patients, the combined model had the highest diagnostic performance for multiple HPV infections. Circulating miRNA signatures show promise as liquid biopsy biomarkers for detecting cervical dysplasia and stratifying for HPV status in HSIL, warranting validation in larger prospective studies.

## 1. Introduction

Cervical carcinogenesis is marked by persistent infection with high-risk genotypes of the sexually transmitted human papillomavirus (hr-HPV) in conjunction with multiple genetic and epigenetic aberrations, which accumulate over several years to decades [1]. The mostly asymptomatic advance from high-grade squamous cell intraepithelial lesions (HSIL) to invasive carcinoma renders cervical cancer accessible to secondary prevention, which is especially important during precancerous stages. Recommended screening tests encompass hr-HPV testing and often cytology, followed by visual inspection with acetic acid and cervical biopsy in cases with abnormal results [2].

Over the past decades, both primary and secondary preventative strategies have proven effective in reducing the incidence and mortality of cervical cancer [3,4]. Yet despite these medical advances, the global burden of disease still designates cervical cancer a public health problem of global proportions: According to the latest estimates, it remains the fourth most common malignant tumor diagnosis for women globally, with 661,021 new cases and 348,189 deaths recorded in 2022. Additionally, existing disparities in adequate health care create vast regional variance in incidence and mortality rates, with up to 10-fold differences between nations on both extremes of the Human Development Index [5]. Unfortunately, projections for 2050 estimate further increases in incidence and mortality at even greater global inequality [6].

As a response, the World Health Organization (WHO) released its global strategy for an accelerated cervical cancer elimination in 2020, hallmarked by a vaccination coverage of 90%, a secondary prevention coverage with a high-performance test of 70%, and treatment coverage of 90% for women with cervical cancer and precancer [7]. Multiple studies cite social, cultural, societal, and structural barriers to screening uptake [8,9], whilst vaccine hesitancy and logistic and financial factors, amongst others, contribute to low vaccination rates, which are estimated to range between 46% and 71% for low-income and high-income countries [10,11,12].

According to current epidemiological models, LSIL, HSIL, and invasive cancer will continue to occur, putting even greater emphasis on highly efficient screening methods, of which current methods fall short [13]. An ideal diagnostic tool combines high sensitivity and specificity, low invasiveness, high accessibility, and ease of implementation, possibly even by laypersons, and in low-resource settings. Liquid biopsy, requiring merely a small sample of bodily fluids for biomarker quantification, has consecutively become a promising focus of research for high-performance tests. This is especially true for microRNAs (miRNAs), small non-coding RNAs central in the regulation of gene transcription. By inhibiting the transcription of tumor suppressors or oncogenes, they themselves become promoters of or barriers to carcinogenesis, with their concentrations correlating with complex intracellular pathobiology. Since they are also secreted from cells and detectable in multiple bodily fluids, most importantly blood, they hold the potential to become highly sensitive non-invasive markers of cancer development and progression [14,15,16].

This retrospective study sought to quantify the expression of five miRNAs (miR-21, miR-142, miR-145, miR-205, and miR-218) and analyze their diagnostic performance individually and in combination for the diagnosis of HSIL at high risk of progression into invasive cervical carcinoma. The miRNA selection is based on evidence of differential expression in cervical neoplasias in previous liquid biopsy studies [17,18,19]. Furthermore, this study evaluates the relationship between miRNA expression and hr-HPV status.

## 2. Materials and Methods

### 2.1. Study Design

For the selection of miRNAs and an endogenous control, a literature search was conducted on Pubmed from July 2021 to August 2021 using the following search terms: <mirna AND cervical cancer AND plasma>; <mirna AND cervical lesion AND plasma>; <mirna AND CIN3 AND plasma>. Only circulating miRNA results of studies indicating significant dysregulation in precancerous lesions or cervical cancer were included, and all those with inconclusive or contradictory results were excluded, leading to the final selection of miR-21-5p, miR-142-3p, miR-145-5p, miR-205-5p, and miR-218-5p, as well as miR-23a-3p as the endogenous control. A synopsis of the results of the literature research can be found in the Supplementary Materials [17,18,19,20,21,22,23,24,25,26,27,28,29,30,31,32,33,34,35,36]. The cohort design of this retrospective study comprised three groups: healthy controls, patients with pathologically confirmed high-grade squamous cell intraepithelial lesions (HSIL), and patients with pathologically confirmed invasive cervical cancer. Prior to enrolment, the sample size was estimated using PASS software Version 21.0.2 (NCSS, Kaysville, UT, USA) for receiver operating characteristic (ROC) curve analysis. Due to the low local cervical cancer prevalence, only HSIL and healthy cohorts were considered. Assuming an expected area under the curve (AUC) of 0.70 compared with the null hypothesis value of 0.50, a two-sided significance level of α = 0.05, and a case–control ratio of approximately 1:1, a total sample size of 70 subjects (32 controls and 38 HSIL cases) was required to achieve 89% power. The primary study endpoint was the differentiation of disease status by selected miRNAs. Further candidate variables examined included recurrence of dysplasia after conization, postoperative HPV status, and Pap smear results for HSIL patients for exploratory analyses.

### 2.2. Sample Acquisition

This study was conducted in accordance with the Declaration of Helsinki and granted approval by the Ethics Committee of the Medical Association of Hamburg (Ethik-Kommission der Ärztekammer, Hamburg, Germany), Number PV5392, on 6 December 2016, with the latest amendment Number PV5392-3704-BO-ff approved on 9 November 2023. A total of 80 EDTA whole-blood samples collected at the University Clinic Hamburg-Eppendorf between April 2021 and April 2022 were included in the study. These include 38 samples from patients with HSIL (CIN 2–3), 32 samples from age-matched healthy female controls, and ten samples from patients with invasive cervical cancer. HSIL and cervical cancer patients were selected from internal databases of the Department of Tumor Biology, which had recruited patients with cytology results indicative of HSIL (CIN 2–3) lesions treated at the Department of Gynecology, and cervical cancer patients undergoing treatment at the Department of Radiotherapy and Radiation Oncology were also selected. Inclusion criteria for HSIL patients were a histologically confirmed diagnosis of CIN2 or CIN3 based on tissue samples acquired during conization, informed written consent, conization for treatment, and availability of clinical data. Inclusion criteria for cervical cancer patients were a histologically confirmed invasive cervical cancer diagnosis and written informed consent. Exclusion criteria for both HSIL and cervical cancer patients were an insufficient amount of biomaterial, different diagnoses, second primary malignancy, and lack of clinical data. The samples for healthy female controls were donated by women without known underlying conditions who were recruited during routine blood donation at the Department of Transfusion Medicine and met health eligibility criteria for blood donation. All subjects gave their written and informed consent before participating in this study. Blood samples for HSIL patients were acquired the day of conization before the procedure took place; samples for cervical cancer patients were obtained during chemotherapy (*n* = 6), prior to surgery (*n* = 3), or before radiotherapy (*n* = 1). HSIL patients were contacted for follow-up information after conization between March 2024 and March 2026 for a mean follow-up time of 18 months. A patient flowchart can be found in the Supplementary Materials.

### 2.3. Plasma Isolation from EDTA Whole Blood

After blood withdrawal, ethylenediaminetetraacetic acid (EDTA) whole-blood samples underwent double blinding for anonymization and were processed within two hours under sterile conditions. Plasma was separated from cellular components by two centrifugation steps with discard of cellular residue (ten minutes at 1900× *g*, ten minutes at 16,000× *g*). The remaining supernatant was analyzed visually for hemolysis, aliquoted into fresh vials, and either processed directly or stored at −80 °C until further usage.

### 2.4. RNA Extraction and Reverse Transcription

Isolation of circulating RNA from plasma was performed with the ‘miRNeasy Serum/Plasma Advanced Kit’ (QIAGEN, Hilden, Germany, Cat. No. 217204) according to the manufacturer’s protocol. Additionally, a ‘miRCURY RNA Spike-In Kit, for RT’ (QIAGEN, Cat. No. 339390) was used for quality control. All components were stored and prepared as recommended. All steps were executed at room temperature unless otherwise stated. The final eluate was aliquoted and stored at −80 °C until further usage. For the retro-transcription of RNA into cDNA, the ‘miRCURY LNA RT Kit’ (QIAGEN, Cat. No. 339340) was used according to the manufacturer’s protocol. All components were stored at −20 °C. In total, 8 µL of the master mix solution was pipetted into 200 µL tubes, and 2 µL of the RNA sample was added. The vials were shaken gently to allow mixing of the reaction solution and RNA sample, and they were shortly centrifuged. Then, they were placed inside a thermocycler, which was programmed according to the reverse transcription temperature protocol. After reverse transcription was complete, all samples were either processed immediately or stored at −20 °C until further use.

### 2.5. Real-Time Quantitative Polymerase Chain Reaction (RT-qPCR)

For quantification of miRNA expression, RT-qPCR was performed using the ‘miRCURY LNA SYBR^®^ Green PCR Kit’ (QIAGEN, Cat. No. 339346) and primers (QIAGEN, Cat. No. 339306) as listed in Table S5 according to the manufacturer’s protocol. All reagents were stored and prepared as instructed by the manufacturer and thawed and kept on crushed ice with samples during preparation. The cDNA samples were diluted 1:20 with RNase-free water. Then, 7 µL of the reaction solution and 3 µL of the diluted cDNA were added to the wells of a 96-well flat, clear-bottom PCR plate. Samples were set up in triplicate for each primer (Supplementary Table S5). Before analysis in a C1000 Touch Thermal Cycler (Bio-Rad Laboratories Inc., Hercules, CA, USA, Cat. No. 1851196) with a CFX96 Touch Real-Time PCR Detection System (Bio-Rad Laboratories Inc., Cat. No. 1845097), the PCR plates were shortly centrifuged. The cycler algorithm was performed according to the following program: initial heat activation for 2 min at 95 °C, followed by a 2-step cycle sequence consisting of denaturation at 95 °C for 10 s and a combined annealing and extension phase at 56 °C for 60 s. Both steps were repeated for a total of 40 cycles. Data acquisition was performed at the end of each cycle. If loaded PCR plates were not immediately analyzed, they were stored at 4 °C for a maximum of six hours.

### 2.6. Statistical Analysis

All statistical analyses were performed with IBM SPSS Statistics for Mac, Version 28.0 (SPSS Inc., IBM Corp., Armonk, NY, USA). Patient data were acquired by clinicians of the Department of Gynecology, transferred to the laboratory, and anonymized for analysis. Quantification cycle (Cq) values derived from RT-qPCR were normalized to the endogenous control miRNA-23a-3p, and relative expression levels were calculated using the ΔCT method. ΔCT-values were subsequently used for all statistical analyses. One sample from the healthy control group with missing miR-218 measurements due to failed amplification was excluded from analyses involving miR-218, but it was retained for analyses of other markers where data were available.

Normal distribution of miRNA expression was assessed visually using histograms and Q-Q plots. Differences in miRNA expression between study groups, as well as between HPV status (HPV16/18 positive vs. negative and single vs. multiple HPV genotype positivity) within the HSIL cohort, were compared using Welch’s *t*-test. Binary logistic regression with additional age-adjusted analyses was performed to estimate the association between miRNA expression levels and disease status or HPV status within the HSIL cohort. The diagnostic performance of individual miRNAs and miRNA panels was assessed by receiver operating characteristic (ROC) curve analysis, and for the latter, the analysis was based on predicted probabilities derived from multivariable logistic regression models. The area under the curve (AUC) was calculated with 95% confidence intervals, and optimal cutoffs were determined using the Youden Index. For disease status discrimination, restrained optimization was used by only considering thresholds yielding a sensitivity of at least 70%, and the cutoff with the highest Youden index within this range was selected. No optimization was applied to cutoffs for HPV stratification.

All statistical tests were two-sided, and *p*-values ≤ 0.05 were considered statistically significant. All graphs were generated using GraphPad Prism 11 (GraphPad Software, Boston, MA, USA).

## 3. Results

### 3.1. Study Population

A total of 80 women were included in the study, comprising 32 healthy controls, 38 high-grade squamous cell intraepithelial lesion (HSIL) patients confirmed by targeted biopsy, and 10 cervical cancer patients (tumor stages FIGO IB2-IIIC2). The control group included female blood donors, and all samples of HSIL patients were collected prior to cervical conization at the University Medical Center Hamburg-Eppendorf. High-risk human papillomavirus (hr-HPV) positivity in HSIL and cervical cancer groups was 95% and 71%. In total, 45% of HSIL patients tested positive for HPV16/18, 40% of the HSIL patients had multiple infections, and two HSIL patients were negative for any HPV genotype. Patient characteristics of the HSIL cohort are summarized in Table 1. Further characterization of the cervical cancer group and hr-HPV distribution in the HSIL group, both pre- and post-operatively, can be found in Supplementary Tables S1 and S2. Whereas there was no significant age-difference between healthy controls (mean age ± standard deviation = 40.8 ± 12.9 years) and HSIL patients (39.3 ± 8.6 years), the cervical cancer patients were slightly older (51.4 ± 12.1). Three cases with persistent hr-HPV infection were detected among the HSIL cohort over a mean follow-up period of 18 months, of which two patients developed recurrent dysplasia (LSIL). The majority of HSIL patients (*n* = 18) showed non-pathological Pap smear results (Pap I) after surgery over the follow-up period.

### 3.2. Differential miRNA Expression Across Study Groups

In an interim analysis of 29 patients (17 HSIL, seven controls, and five cancer), miR-142 and miR-145 did not exhibit statistically significant differential expression between HSIL and control groups and were therefore excluded from the final analysis comprising the complete cohort (Supplementary Figure S1).

**Figure 1:**
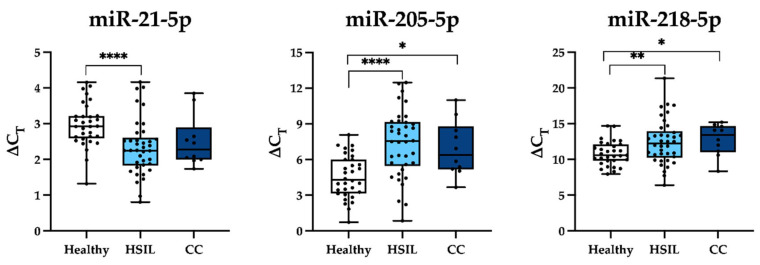
Expression patterns of individual miRNAs in healthy controls, HSIL patients, and cervical cancer (CC) patients. miRNA expression is presented as ΔCT values. Statistical significance of differential expression between groups was assessed using Welch’s *t*-test. Significance levels are indicated as follows: *p* < 0.05 (*), *p* < 0.01 (**), and *p* < 0.0001 (****).

In the final analysis, miR-21 expression was significantly upregulated in HSIL patients relative to healthy controls, whereas miR-205 and miR-218 were significantly downregulated in both HSIL and cervical cancer cohorts (Figure 1). No statistically significant differences were observed between HSIL and cervical cancer patients. The mean expression levels and corresponding *p*-values are shown in Supplementary Table S3.

### 3.3. Association Between miRNA Expression and Disease Status

Binary logistic regression analysis was used to assess associations between disease status and miRNA levels with significant differential expression. Expression levels of all three miRNAs (miR-21, miR-205, and miR-218) were significantly associated with HSIL status when compared with healthy controls (Table 2).

For cervical cancer patients, differential expression of miR-205 and miR-218 was significantly associated with disease status, whereas miR-21 was not significantly associated with cervical cancer in either unadjusted or age-adjusted models. Detailed results of logistic regression analyses are presented in Table 3. Since a significant age difference between the cervical cancer and the healthy control cohort was detected (*p* = 0.04), age-adjusted analyses were included. Age-adjusted analyses yielded comparable odds ratios and *p*-values.

### 3.4. Diagnostic Performance of Individual miRNAs and miRNA Panels

To assess the diagnostic performance of the three individual miRNAs and miRNA panels based on significantly dysregulated miRNAs for distinguishing healthy controls from HSIL and cervical cancer patients, receiver operating characteristic (ROC) curve analysis was used (Table 4).

**Figure 2:**
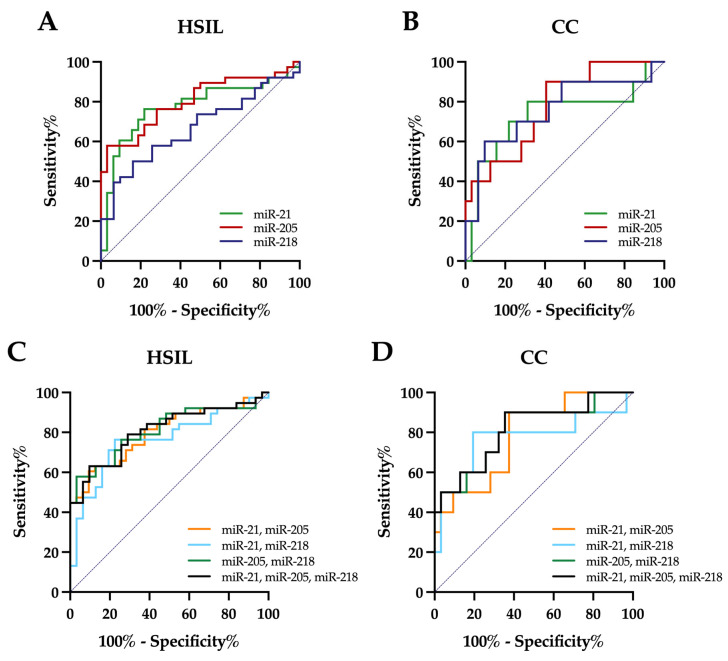
ROC curves illustrating the diagnostic performance of circulating miRNAs for distinguishing healthy controls from HSIL and cervical cancer (CC) patients. Panels A and B show ROC curves for individual miRNAs (miR-21, miR-205, and miR-218) for discrimination between healthy controls and HSIL (**A**) and cervical cancer patients (**B**). Panels C and D present ROC curves for multivariable miRNA panels derived from logistic regression models for HSIL (**C**) and cervical cancer (**D**).

For discrimination between healthy controls and HSIL patients, miR-205 achieved the highest area under the curve (AUC) among individual miRNAs (AUC = 0.80, 95% CI 0.70–0.91), with an estimated sensitivity of 76% and specificity of 72%. miR-21 showed a similar sensitivity with a slightly higher specificity (78%) and a comparable AUC (0.78, 95% CI 0.66–0.89). ROC curves are shown in Figure 2.

Combining miR-21 and miR-205 resulted in a similar AUC (0.80, 95% CI 0.69–0.90). The highest AUC values were observed for the two-miRNA panel consisting of miR-205 and miR-218 and for the three-miRNA panel (miR-21, miR-205, and miR-218), both yielding an AUC of 0.81 (95% CI 0.71–0.91), as shown in Figure 2.

For discrimination between healthy controls and cervical cancer, miR-205 equally achieved the highest AUC among individual miRNAs (AUC = 0.78, 95% CI 0.62–0.94) with a sensitivity of 90% and a specificity of 59%. An increase in AUC to 0.81 was observed when combining miR-205 with miR-218 in a two-miRNA panel (95% CI 0.64–0.97) or with miR-21 and miR-218 in a three-miRNA panel (95% CI 0.65–0.98), with a sensitivity of 90% and a specificity of 65%. More details are presented in Table 4 and Figure 2.

### 3.5. miRNAs in HPV Stratification—Association Between miRNA Expression and HPV Status and Diagnostic Performance in HSIL

Within the HSIL cohort, expression of significantly dysregulated miRNAs was further evaluated in relation to HPV status (Table 5). miR-205 expression differed significantly between HPV16/18-positive (*n* = 18) and HPV16/18-negative patients (*n* = 20). HPV16/18 positivity was associated with higher expression levels (OR 0.67, 95% CI 0.49–0.92, *p* = 0.013). Lower miR-218 expression was significantly associated with the presence of multiple HPV genotypes (≥2 genotypes, *n* = 15) compared with a single HPV genotype (*n* = 21) (OR 1.32, 95% CI 1.02–1.72, *p* = 0.039). Further details about miRNA expression levels can be found in Supplementary Table S4.

Assessments of the diagnostic performance of individual miRNAs and miRNA panels distinguishing HPV status showed that among all individual miRNAs, as well as combined miRNA panels, miR-205 had the highest AUC of 0.77 (95% CI 0.62–0.92), directly followed by any combined model of miR-205 with the other miRNAs (AUC 0.76, 95% CI 0.61–0.92, each) (Table 6). For single versus multiple infections, miR-218 yielded the highest AUC among individual miRNAs (AUC = 0.69, 95% CI 0.51–0.87), whereas the highest AUC overall of 0.84 (95% CI 0.71–0.97) was achieved by a combination of all investigated miRNAs (miR-21, miR-205, and miR-218) (Table 6). Low sample sizes (*n* ≤ 7) in the other most common hr-HPV genotype subgroups (HPV31, HPV52, and HPV58) among the HSIL cohort precluded meaningful statistical analyses.

## 4. Discussion

In this retrospective study, five circulating miRNAs derived from blood samples were evaluated for their diagnostic potential in distinguishing HSIL and cervical cancer from healthy controls in a liquid biopsy approach individually and in panels. Of the five assessed miRNAs, three (miR-21, miR-205, and miR-218) proved to be significantly dysregulated among our study groups. miR-21 expression was significantly upregulated in HSIL patients, whereas miR-205 and miR-218 were significantly downregulated in both HSIL and cervical cancer patients. Logistic regression and ROC curve analyses indicated that miR-205 showed the strongest diagnostic performance among individual miRNAs. Combining miRNAs in a three-marker panel resulted in a meaningful improvement in diagnostic discrimination. In exploratory subgroup analyses within the HSIL cohort, high miR-205 expression was associated with HPV16/18 positivity, whereas lower miR-218 expression was associated with the presence of multiple HPV genotypes. As for the disease status, the three-marker panel signature yielded an improved diagnostic distinction between single and multiple HPV genotypes. This may imply that a higher viral load is associated with a stronger signature and vice versa, requiring closer clinical surveillance due to an increased risk of progression.

In our study, miR-21 expression was significantly upregulated in HSIL patients compared with healthy controls, supporting its potential role in early cervical carcinogenesis. miR-21 is widely recognized as an oncogenic miRNA and has been reported to be overexpressed across multiple tumor entities. In cervical cancer specifically, miR-21 has been shown to promote tumor progression by targeting several tumor suppressor genes, including PTEN, RECK, and NTF3, thereby enhancing cellular proliferation, migration, and invasion [37,38,39,40]. Increased miR-21 expression has also been observed during the progression from normal cervical epithelium to HSIL and invasive cancer in previous studies [41,42]. Together, these findings suggest that elevated circulating miR-21 levels may reflect early molecular alterations associated with HPV-driven cervical transformation.

While miR-21 represents a well-established oncogenic miRNA involved in cervical tumor development, our analyses indicated that miR-205 showed the strongest diagnostic performance among the investigated biomarkers. In the present study, miR-205 was significantly downregulated in HSIL and cervical cancer patients compared with healthy controls, supporting its potential role as a biomarker for HSIL detection. miR-205 has been described as a context-dependent regulator in carcinogenesis, with studies reporting both tumor-suppressive and oncogenic functions [31,43]. In several malignancies, including melanoma, renal cancer, esophageal cancer, and triple-negative breast cancer, miR-205 has been proven to act as a tumor suppressor by inhibiting proliferation, migration, and epithelial–mesenchymal transition through targets such as E2F1, ZEB1, and ZEB2 [43,44]. In contrast, pro-tumorigenic effects have been observed in other tumor entities, including colorectal, nasopharyngeal, endometrial, and ovarian cancers, where miR-205 promotes cell proliferation and survival through pathways involving PTEN [45,46].

In cervical carcinogenesis, previous studies have similarly reported both the up- and downregulation of miR-205 in precancerous lesions and invasive cervical cancer [47,48,49,50], suggesting a complex and potentially stage-dependent regulatory role. These divergent findings may reflect differences in the tumor microenvironment, HPV-related molecular alterations, or context-dependent regulation of miR-205 target genes. The downregulation of miR-205 observed in our study is therefore consistent with reports suggesting a tumor-suppressive role of miR-205 in cervical epithelial transformation.

Consistent with our findings, miR-218 has frequently been reported to be downregulated during carcinogenesis across multiple tumor entities, including cervical cancer [51,52,53,54]. miR-218 is considered a tumor suppressor miRNA and has been implicated in regulating key processes such as cell proliferation, migration, and apoptosis [52,53,54]. Several oncogenic signaling pathways have been reported to be influenced by miR-218, including the AKT/mTOR, wnt/ß-catenin, and NF-κB pathways. In addition, miR-218 targets multiple genes involved in tumor progression, including survivin and RUNX2 [55,56]. Notably, HPV infection itself has been shown to contribute to reduced miR-218 expression through activity of the viral oncoproteins E6 and E7 [57,58], providing a potential mechanistic explanation for the decreased expression observed in cervical dysplasia and cervical cancer in our study.

Regarding the diagnostic performance of miRNAs, combining the significantly dysregulated markers into a multi-miRNA panel led to a noticeable enhancement in diagnostic discrimination. Similar observations have been reported in several studies evaluating circulating miRNA biomarkers in cervical cancer, where panels consisting of multiple dysregulated miRNAs often outperform single markers [59]. This improvement may reflect the complex regulatory nature of miRNAs, as multiple miRNAs can influence overlapping signaling pathways and cellular processes involved in tumorigenesis. Consequently, integrating several miRNAs may prove a more comprehensive representation of disease-associated molecular alterations than individual markers alone. Recent studies have further suggested that such regulatory networks may vary across different stages of cervical carcinogenesis, highlighting the potential value of multi-marker approaches in diagnostic biomarker development [54,55,60,61]. One limitation of our study is the small sample size, which necessitates cautious interpretation of results and affects transferability to the entire population of interest. To achieve a more pronounced increase in AUC than observed in our study, larger panels or additional biomarkers may be required to substantially enhance diagnostic performance. Furthermore, the study design does not allow conclusions regarding temporal changes in miRNA expression during disease progression. Further studies with greater sample sizes and prospective designs are needed to confirm the diagnostic value of the investigated miRNAs.

Exploratory analyses within the HSIL cohort indicated associations of miR-205 expression with HPV16/18 positivity and low miR-218 levels with multi-genotype HPV infection. Previous studies have provided insights into how HPV oncoproteins might influence miRNA expression patterns and contribute to HPV-driven carcinogenesis [60]. HPV oncoproteins E6 and E7 have been shown to modulate the expression of both oncogenic and tumor suppressor microRNAs [62]. The more pronounced decrease in miR-218 in multiple HPV infections likely reflects an increased cumulative burden of viral oncoproteins and a more advanced, persistent lesion biology, leading to the stronger repression of this tumor-suppressive pathway. Although the limited sample size requires cautious interpretation, our findings suggest that HPV genotype-specific molecular alterations may partially contribute to the differential expression of circulating miRNAs observed in cervical dysplasia.

Circulating miRNAs have emerged as promising candidates for non-invasive cancer diagnostics due to their stability in blood, resistance to enzymatic degradation, and their involvement in key regulatory pathways of carcinogenesis [15]. In cervical cancer screening, liquid biopsy approaches may provide molecular information mirroring early cellular alterations associated with HPV-driven carcinogenesis and thereby complement and refine existing diagnostic methods. Cervical sampling, including HPV genotyping and methylation analysis, remains highly relevant for HSIL management. Additionally, the increasing investigation of circulating exosomal miRNAs, which is currently still limited by a lack of standardization, may in the future provide more tumor-cell-specific insights. In this setting, circulating miRNA analysis is meant to provide a complementary approach rather than a replacement for cervical testing. Its value lies in adding systemic biomarker information.

Consequently, identifying circulating miRNA signatures may contribute to improved risk stratification of premalignant cervical dysplasia and early detection strategies before progression into invasive cervical carcinoma. Integrating miRNA biomarkers with other molecular or clinical parameters or using longitudinal designs may further enhance diagnostic accuracy. Comparing biomarker performance with local HPV detection methods reflective of biological HPV status may also provide new aspects for clinical applicability. Furthermore, mechanistic studies exploring the interaction between HPV infection and miRNA regulatory networks may improve our understanding of the molecular processes underlying cervical carcinogenesis and support the development of novel diagnostic strategies. Together, such approaches may help advance the development of minimally invasive biomarkers for the detection and monitoring of cervical disease.

Our minimally invasive liquid biopsy approach based on circulating miRNAs, together with the integrated application of complementary statistical methods, enables a comprehensive evaluation of the diagnostic performance of relevant biomarkers in cervical carcinogenesis. The inclusion of HPV stratification analyses provides additional insight into potential virus-associated molecular alterations affecting miRNA expression.

Overall, circulating miRNA signatures represent a promising minimally invasive approach for improving molecular detection of cervical dysplasia and warrant further investigation in larger clinical studies.

## 5. Conclusions

In summary, this study showed the improved diagnostic performance of a three-miRNA signature based on circulating miRNAs in detecting HSIL compared to individual biomarkers, as well as the potential of miRNAs to distinguish between HPV status, which might reflect HPV-related molecular alterations relevant to risk stratification. Further large-scale studies are needed to investigate their potential application in early detection and disease monitoring.

## Figures and Tables

**Table 1:** Clinicopathological characteristics of HSIL patients (*n* = 38).

Age, *Years Mean ± SD*		39.3 ± 8.6
hr-HPV *n* (%)	*Any type*	36 ^#^ (95)
	*HPV16/18*	18 (47)
	*Non-HPV16/18*	25 (66)
	*Single HPV genotype*	21 (55)
	*≥2 HPV genotypes*	15 (40)
	No Data *	-
Vaccinated *n* (%)	*Yes*	2 (7)
	*No*	27 (93)
	No Data *	9
Recurrence ^§^ *n* (%)	*Yes*	2 (7)
	*No*	25 (93)
	No Data *	11
Postoperative Pap results *n* (%)	*I*	18 (62)
	*IIa*	8 (28)
	*IIp*	2 (7)
	*IIID1*	1 (3)
	No Data *	9

* For reasons of statistical clarity, missing data is reported as part of 100%. ^#^ Two HSIL patients were tested negative for hr-HPV at the time of blood withdrawal. ^§^ Mean follow-up time of 18 months (range 5–40 months).

**Table 2:** Association between miRNA expression levels and disease status for HSIL patients compared to healthy controls.

	miRNA	OR	95% CI	*p*-Value
**Healthy vs. HSIL**	miR-21	0.28	0.13–0.62	0.002
	miR-205	1.65	1.27–2.15	<0.001
	miR-218	1.31	1.05–1.64	0.018

Odds ratios (ORs) with 95% confidence intervals (CIs) and *p*-values were derived from binary logistic regression analyses.

**Table 3:** Association between miRNA expression levels and disease status for cervical cancer patients compared to healthy controls, including age-adjusted and unadjusted results.

	*miRNA*	Not Age-Adjusted			Age-Adjusted		
		*OR*	*95% CI*	*p-Value*	*OR*	*95% CI*	*p-Value*
**Healthy vs. CC**	miR-21	0.29	0.08–1.06	0.061	0.22	0.05–1.02	0.053
	miR-205	1.85	1.16–2.93	0.009	2.17	1.20–3.81	0.010
	miR-218	1.73	1.12–2.68	0.013	2.18	1.22–3.90	0.009

Odds ratios (ORs) with 95% confidence intervals (CIs) and *p*-values were derived from binary logistic regression analyses with and without age adjustment.

**Table 4:** ROC curve analysis evaluating the diagnostic performance of individual miRNAs and miRNA panels for distinguishing healthy controls from HSIL and cervical cancer patients.

	miRNAs	AUC	95% CI	*p*-Value	Sensitivity	Specificity	Cutoff *
**Healthy vs. HSIL**	miR-21	0.78	0.66–0.89	<0.001	76%	78%	ΔC_T_ = 2.57
	miR-205	0.80	0.70–0.91	<0.001	76%	72%	ΔC_T_ = 5.57
	miR-218	0.67	0.54–0.80	0.010	74%	52%	ΔC_T_ = 10.63
	miR-21 and miR-205	0.80	0.69–0.90	<0.001	71%	72%	PP = 0.53
	miR-21 and miR-218	0.76	0.65–0.88	<0.001	76%	77%	PP = 0.55
	miR-205 and miR-218	0.81	0.71–0.91	<0.001	76%	74%	PP = 0.52
	miR-21, miR-205 and miR-218	0.81	0.71–0.91	<0.001	79%	71%	PP = 0.48
**Healthy vs. CC**	miR-21	0.73	0.52–0.94	0.031	70%	78%	ΔC_T_ = 2.56
	miR-205	0.78	0.62–0.94	0.001	90%	59%	ΔC_T_ = 5.02
	miR-218	0.76	0.57–0.95	0.007	70%	74%	ΔC_T_ = 11.92
	miR-21 and miR-205	0.78	0.62–0.94	0.008	90%	63%	PP = 0.18
	miR-21 and miR-218	0.77	0.56–0.98	0.011	80%	81%	PP = 0.27
	miR-205 and miR-218	0.81	0.64–0.97	0.004	90%	65%	PP = 0.16
	miR-21, miR-205 and miR-218	0.81	0.65–0.98	0.003	90%	65%	PP = 0.17

The table shows the area under the curve (AUC) with 95% confidence intervals (CIs), *p*-values, and optimal cutoffs with corresponding sensitivity and specificity. * Cutoffs are presented as ΔCT values for individual miRNA and predicted probability (PP) derived from multivariable logistic regression analysis for miRNA panels. Optimal cutoffs were determined using the Youden Index after constrained optimization for cutoffs with a sensitivity of ≥70%.

**Table 5:** Association between miRNA expression levels and HPV16/18 positivity and HPV count in the HSIL subgroup.

	miRNA	OR	95% CI	*p*-Value
**HPV16/18-positive vs. -negative**	miR-21	2.27	0.88–5.85	0.088
	miR-205	0.67	0.49–0.92	**0.013**
	miR-218	0.85	0.68–1.07	0.171
**Single vs. ≥2 HPV-positive**	miR-21	0.72	0.30–1.75	0.472
	miR-205	0.96	0.76–1.23	0.762
	miR-218	1.32	1.02–1.72	**0.039**

Odds ratios (ORs) with 95% confidence intervals (CIs) and corresponding *p*-values were derived from binary logistic regression analyses. Significant *p*-values in bold.

**Table 6:** ROC curve analysis evaluating the diagnostic performance of individual miRNAs and miRNA panels for distinguishing HPV status within the HSIL cohort.

	miRNAs	AUC	95% CI	*p*-Value	Sensitivity	Specificity	Cutoff *
**HPV16/18-positive vs. -negative**	miR-21	0.68	0.51–0.85	0.035	94%	40%	ΔC_T_ = 1.81
	miR-205	0.77	0.62–0.92	<0.001	94%	55%	ΔC_T_ = 8.75
	miR-218	0.62	0.43–0.80	0.094	67%	65%	ΔC_T_ = 12.25
	miR-21 and miR-205	0.76	0.61–0.92	0.005	94%	50%	PP = 0.33
	miR-21 and miR-218	0.65	0.47–0.83	0.114	94%	50%	PP = 0.39
	miR-205 and miR-218	0.76	0.61–0.92	0.006	83%	65%	PP = 0.41
	miR-21, miR-205 and miR-218	0.76	0.61–0.92	0.006	83%	65%	PP = 0.41
**Single vs. ≥2 HPV-positive**	miR-21	0.59	0.39–0.79	0.395	47%	81%	ΔC_T_ = 1.94
	miR-205	0.50	0.28–0.73	0.989	47%	86%	ΔC_T_ = 8.86
	miR-218	0.69	0.51–0.87	0.044	53%	91%	ΔC_T_ = 13.60
	miR-21 and miR-205	0.60	0.41–0.79	0.312	80%	48%	PP = 0.38
	miR-21 and miR-218	0.71	0.52–0.89	0.038	60%	86%	PP = 0.51
	miR-205 and miR-218	0.82	0.68–0.96	0.001	80%	71%	PP = 0.37
	miR-21, miR-205, and miR-218	0.84	0.71–0.97	0.001	80%	76%	PP = 0.40

The table shows the AUC with 95% confidence intervals (CIs), *p*-values, and optimal cutoffs with corresponding sensitivity and specificity as determined using the Youden Index. * Cutoffs are presented as ΔCT values for individual miRNA and predicted probability (PP) derived from multivariable logistic regression analysis for miRNA panels.

## Data Availability

The raw data supporting the conclusions of this article will be made available by the authors upon request.

## Supplementary Materials

The following supporting information can be downloaded at https://www.mdpi.com/article/10.3390/cells15090849/s1. Figure S1: Expression patterns of individual miRNAs in healthy controls, HSIL patients, and cervical cancer patients in a first analysis; Table S1: Clinicopathological characteristics of cervical cancer patients, including FIGO stage and carcinoma type; Table S2: Pre- and postoperative hr-HPV status of HSIL patients. Table S3: Differential miRNA expression across study groups; Table S4: Differential miRNA expression according to hr-HPV status among the HSIL cohort; Table S5: Summary of primers used in RT-qPCR [17,18,19,20,21,22,23,24,25,26,27,28,29,30,31,32,33,34,35,36].

## Abbreviations

The following abbreviations are used in this manuscript:

AKT	Protein kinase B;
AUC	Area under the curve;
CC	Cervical cancer;
CI	Confidence interval;
CIN	Cervical intraepithelial lesion;
cDNA	Complementary deoxyribonucleic acid;
DNA	Deoxyribonucleic acid;
E2F1	E2F transcription factor 1;
EDTA	Ethylenediaminetetraacetic acid;
HPV	Human papillomavirus;
HSIL	High-grade squamous cell intraepithelial lesion;
hr-HPV	High-risk human papillomavirus;
LSIL	Low-grade squamous cell intraepithelial lesion;
NF-κB	Nuclear factor kappa-light-chain-enhancer of activated B cells;
NTF3	Neurotrophin 3;
miRNA	MicroRNA;
mTOR	Mechanistic target of rapamycin;
PTEN	Phosphatase and tensin homolog;
OR	Odds ratio;
RECK	Reversion-inducing cysteine-rich protein with kazal motifs;
RNA	Ribonucleic acid;
ROC	Receiver operating characteristic;
RT-qPCR	Reverse-transcription quantitative polymerase chain reaction;
RUNX2	Runt-related transcription factor 2;
SD	Standard deviation;
WHO	World Health Organization;
ZEB1/2	Zinc finger E-box-binding homeobox 1/2.
