# Supplementary material for: Circulating microRNA Profiles as Diagnostic Tools for High-Grade Cervical Lesions and HPV Genotype Stratification
Source: Cells. 2026 May 6;15(9):849. doi: 10.3390/cells15090849 (PMC13162731; doi:10.3390/cells15090849)
Supplement: Supplementary file 1 [file cells-15-00849-s001.zip › cells-4281931-supplementary.pdf]

Supplements

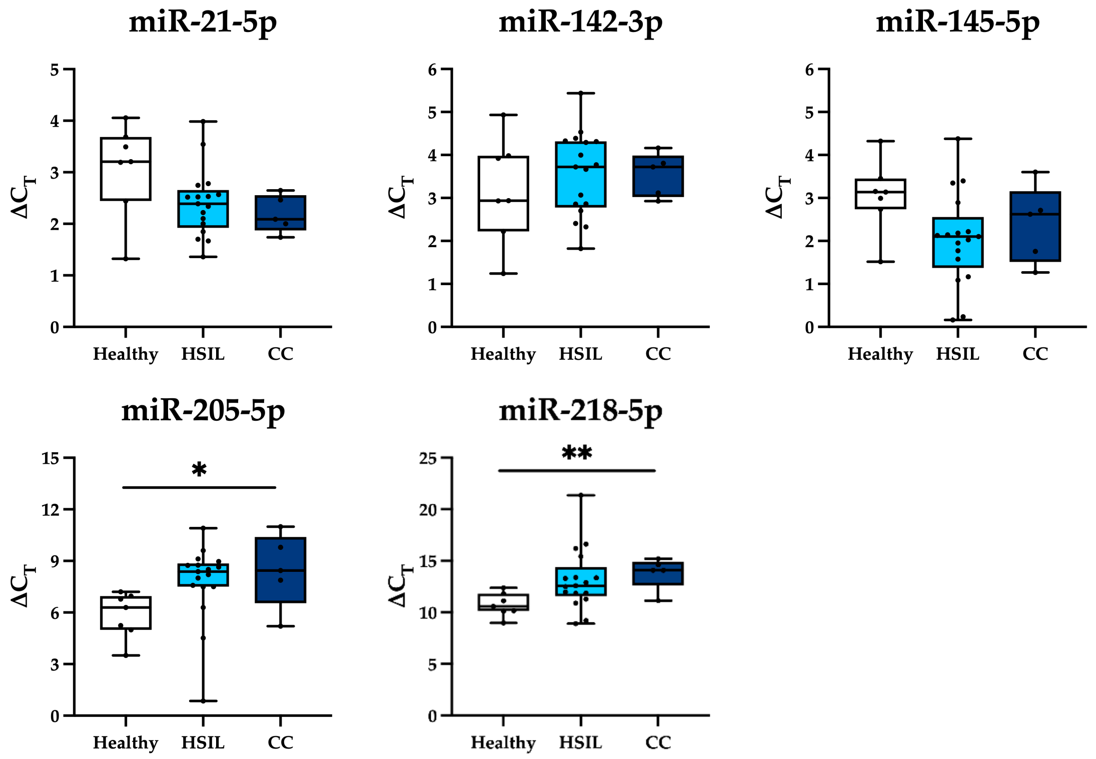

Supplementary Figure S1. Expression patterns of individual miRNAs in healthy controls, HSIL patients, and cervical cancer patients in an interim analysis. miRNA expression is presented as ΔC_T values. Statistical significance of differential expression between groups was assessed using Welch's ANOVA test. Significance levels are indicated as follows: $p < 0.05$ (*), $p < 0.01$ (**).

Supplementary Table S1. Clinicopathological characteristics of cervical cancer patients (n = 10), including FIGO stage and carcinoma type.

**Age, years** *mean ± SD*		51.4 ± 12.1
**hr-HPV** *n (%)*	*any type*	5 (71)
	HPV16/18	5 (71)
	Non HPV16/18	0
	Single HPV genotype	6 (86)
	≥2 HPV genotypes	0
	*No Data**	3
**FIGO stage** *n (%)*	*IB2*	1 (14)
	IIB	2 (29)
	IIIB	2 (29)
	IIIC1	1 (14)
	IIIC2	1 (14)
	*No Data**	3
**Carcinoma type** *n (%)*	*Squamous Cell Carcinoma*	3 (33)
	Adenocarcinoma	6 (67)

*No Data**	1

*For reasons of statistical clarity, missing data is reported as part of 100%.

Supplementary Table S2. Pre- and postoperative high-risk human papillomavirus status of high-grade squamous cell intraepithelial lesion patients.

	Preoperative	Postoperative
**hr-HPV, *any type*** *n (%)*	36 (95)	3 (11)
**Single hr-HPV** *n (%)*	21 (55)	2 (7)
**≥2 hr-HPV** *n (%)*	15 (40)	1 (4)
**HPV16** *n (%)*	16 (42)	1 (4)
**HPV18** *n (%)*	2 (5)	-
**HPV31** *n (%)*	7 (18)	-
**HPV33** *n (%)*	3 (8)	-
**HPV35** *n (%)*	1 (3)	-
**HPV39** *n (%)*	2 (5)	-
**HPV45** *n (%)*	3 (8)	-
**HPV51** *n (%)*	3 (8)	-
**HPV52** *n (%)*	7 (18)	-
**HPV56** *n (%)*	-	1 (4)
**HPV58** *n (%)*	6 (16)	1 (4)
**HPV59** *n (%)*	1 (3)	-
**HPV66** *n (%)*	3 (8)	2 (7)
No Data*	-	11

*For reasons of statistical clarity, missing data is reported as part of 100%.

Supplementary Table S3. Differential miRNA expression across study groups presented as mean ΔCT ± SD.

miRNA	Healthy	HSIL	CC#	Healthy vs. HSIL *p-value**	Healthy vs. CC# *p-value**	HSIL vs. CC# *p-value**
**miR-21**	2.97 ± 0.61	2.34 ± 0.78	2.5 ± 0.72	< 0.001	0.086	0.549
**miR-205**	4.56 ± 1.80	7.34 ± 2.77	6.92 ± 2.32	< 0.001	0.012	0.635
**miR-218**	10.85 ± 1.70	12.46 ± 3.12	12.78 ± 2.23	0.008	0.026	0.721

**P*-values represent comparisons between healthy controls and HSIL or cervical cancer patients using Welch's t-test. #CC = cervical cancer.

Supplementary Table S4. Differential miRNA expression according to HPV status among the HSIL cohort presented as mean ΔCT ± SD.

	HPV16/18 positive	HPV16/18 negative	Mean difference *(95% CI)*	*p*-value	Single HPV positive	≥2 HPV positive	Mean difference *(95% CI)*	*p*-value
**miR-21**	2.58 ± 0.73	2.13 ± 0.73	-0.45 (-0.95-0.05)	0.076	2.42 ± 0.74	2.23 ± 0.86	0.19 (-0.37-0.75)	0.495
**miR-205**	6.07 ± 2.67	8.48 ± 2.38	2.42 (0.74-4.09)	**0.006**	7.49 ± 1.90	7.21 ± 3.76	0.28 (-1.93-2.49)	0.792

**miR-218**	11.72 ± 2.24	13.13 ± 3.67	1.41 (-0.58-4.40)	0.158	11.61 ± 2.51	13.93 ± 3.47	-2.33 (-4.50-(-0.16))	**0.036**

Supplementary Table S5. Summary of primers used in Reverse-Transcription quantitative Polymerase Chain Reaction.

Name	Cat. no.	Gene Globe ID	Mature miRNA sequence (5′-3′)
**hsa-miR-21-5p**	339306	YP00204230	UAGCUUAUCAGACUGAUGUUGA
**hsa-miR-23a-3p**	339306	YP00204772	AUCACAUUGCCAGGGAUUUCC
**hsa-miR-142-3p**	339306	YP00204291	UGUAGUGUUUCCUACUUUAUGGA
**hsa-miR-145-5p**	339306	YP00204483	GUCCAGUUUUCCCAGGAAUCCCU
**hsa-miR-205-5p**	339306	YP00204487	UCCUUCAUUCCACCGGAGUCUG
**hsa-miR-218-5p**	339306	YP00206034	UUGUGCUUGAUCUAACCAUGU

Patient Flowchart

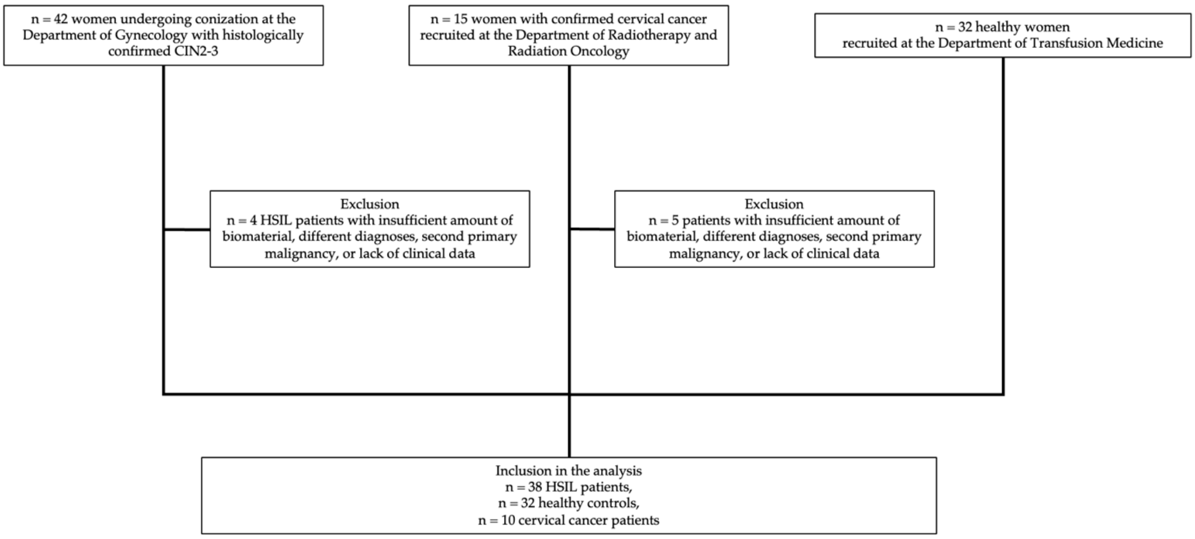

Synopsis of literature research

miR-21-5p: Reported to be upregulated across multiple tumor entities, including cervical cancer, as demonstrated in serum and plasma samples [18, 20, 21].

miR-23a-3p: Demonstrates stable expression in cervical cancer tissue and has commonly been used as an endogenous control for miRNA detection via RT-qPCR [22, 23, 34-36].

miR-142-3p: Exhibits differential expression across various tumor entities, with downregulation in colon cancer [24] and upregulation in head and neck, endometrial, and breast cancer [25-27]. In cervical cancer, it is downregulated and described as a tumor suppressor [28, 29].

miR-145-5p: Widely characterized as a tumor suppressor miRNA across multiple malignancies, including breast, ovarian, lung and colorectal cancer, and reported to be downregulated in plasma samples of cervical cancer patients [18, 20, 30].

miR-205-5p: Displays context-dependent roles across tumor entities, functioning either as tumor suppressor (e.g., in prostate and triple-negative breast cancer) or as an oncogene (e.g., in endometrial and nasopharyngeal cancer) [31]. In cervical cancer, several studies report significant upregulation compared to healthy controls [17, 19].

miR-218-5p: Identified as a tumor suppressor involved in the regulation of proliferation and apoptosis in cervical cancer cells, with significant downregulation in plasma samples of cervical cancer patients [17, 32, 33].

References

17. Ma, Q., G. Wan, S. Wang, W. Yang, J. Zhang and X. Yao, *Serum microRNA-205 as a novel biomarker for cervical cancer patients.* Cancer Cell Int, 2014. **14**: p. 81.
18. Aftab, M., S.S. Poojary, V. Seshan, S. Kumar, P. Agarwal, S. Tandon, V. Zutshi and B.C. Das, *Urine miRNA signature as a potential non-invasive diagnostic and prognostic biomarker in cervical cancer.* Sci Rep, 2021. **11**(1): p. 10323.
19. Farzanehpour, M., S.H. Mozhgani, S. Jalilvand, E. Faghihloo, S. Akhavan, V. Salimi and T.M. Azad, *Serum and tissue miRNAs: potential biomarkers for the diagnosis of cervical cancer.* Virol J, 2019. **16**(1): p. 116.
20. Okoye, J.O., A.A. Ngokere, C.C. Onyenekwe and C.A. Erinle, *Comparable expression of miR-let-7b, miR-21, miR-182, miR-145, and p53 in serum and cervical cells: Diagnostic implications for early detection of cervical lesions.* Int J Health Sci (Qassim), 2019. **13**(4): p. 29–38.
21. Qiu, H., D. Liang, L. Liu, Q. Xiang, Z. Yi and Y. Ji, *A Novel Circulating MiRNA-Based Signature for the Diagnosis and Prognosis Prediction of Early-Stage Cervical Cancer.* Technol Cancer Res Treat, 2020. **19**: p. 1533033820970667.
22. Shen, Y., Y. Li, F. Ye, F. Wang, X. Wan, W. Lu and X. Xie, *Identification of miR-23a as a novel microRNA normalizer for relative quantification in human uterine cervical tissues.* Exp Mol Med, 2011. **43**(6): p. 358–66.
23. Hoelzle, C.R., S. Arnoult, C.R.M. Borem, M. Ottone, K. de Magalhaes, I.L. da Silva and R.T. Simoes, *MicroRNA Levels in Cervical Cancer Samples and Relationship with Lesion Grade and HPV Infection.* Microrna, 2021. **10**(2): p. 139–145.
24. Lee, I.H., G. Kim, S.G. Kwak, D.W. Baek, B.W. Kang, H.J. Kim, S.Y. Park, J.S. Park, G.S. Choi, K. Hur, et al., *Predictive Value of Circulating miRNAs in Lymph Node Metastasis for Colon Cancer.* Genes (Basel), 2021. **12**(2).
25. Summerer, I., K. Unger, H. Braselmann, L. Schuettrumpf, C. Maihoefer, P. Baumeister, T. Kirchner, M. Niyazi, E. Sage, H.M. Specht, et al., *Circulating microRNAs as prognostic therapy biomarkers in head and neck cancer patients.* Br J Cancer, 2015. **113**(1): p. 76–82.
26. Fan, X., M. Cao, C. Liu, C. Zhang, C. Li, W. Cheng, S. Zhang, H. Zhang and W. Zhu, *Three plasma-based microRNAs as potent diagnostic biomarkers for endometrial cancer.* Cancer Biomark, 2021. **31**(2): p. 127–138.
27. Jusoh, A.R., S.V. Mohan, T. Lu Ping, T. Tengku Din, J. Haron, R.C. Romli, H. Jaafar, S.N. Nafi, T.I. Tuan Salwani and M.M. Yahya, *Plasma Circulating Mirnas Profiling for Identification of Potential Breast Cancer Early Detection Biomarkers.* Asian Pac J Cancer Prev, 2021. **22**(5): p. 1375–1381.
28. Li, M., B.Y. Li, H. Xia and L.L. Jiang, *Expression of microRNA-142-3p in cervical cancer and its correlation with prognosis.* Eur Rev Med Pharmacol Sci, 2017. **21**(10): p. 2346–2350.
29. Dong, H. and J. Song, *miR-142-3p reduces the viability of human cervical cancer cells by negatively regulating the cytoplasmic localization of HMGB1.* Exp Ther Med, 2021. **21**(3): p. 212.
30. Wei, H., C. Wen-Ming and J. Jun-Bo, *Plasma miR-145 as a novel biomarker for the diagnosis and radiosensitivity prediction of human cervical cancer.* J Int Med Res, 2017. **45**(3): p. 1054–1060.
31. Ferrari, E. and P. Gandellini, *Unveiling the ups and downs of miR-205 in physiology and cancer: transcriptional and post-transcriptional mechanisms.* Cell Death Dis, 2020. **11**(11): p. 980.
32. Wang, P., G. Zhai and Y. Bai, *Values of miR-34a and miR-218 expression in the diagnosis of cervical cancer and the prediction of prognosis.* Oncol Lett, 2018. **15**(3): p. 3580–3585.
33. Zhang, J., S. Li, Y. Li, H. Liu, Y. Zhang and Q. Zhang, *miRNA-218 regulates the proliferation and apoptosis of cervical cancer cells via targeting Gli3.* Exp Ther Med, 2018. **16**(3): p. 2433–2441.

34. Malta, M., J. Ribeiro, P. Monteiro, J. Loureiro, R. Medeiros and H. Sousa, *Let-7c is a Candidate Biomarker for Cervical Intraepithelial Lesions: A Pilot Study.* Mol Diagn Ther, 2015. **19**(3): p. 191–6.
35. Ribeiro, J., J. Marinho-Dias, P. Monteiro, J. Loureiro, I. Baldaque, R. Medeiros and H. Sousa, *miR-34a and miR-125b Expression in HPV Infection and Cervical Cancer Development.* Biomed Res Int, 2015. **2015**: p. 304584.
36. Coimbra, E.C., D.A.C.G.L. M, M.R. Junior, D.E.O. TH, D.A.C.S.N. J and D.E.F. AC, *Expression Profile of MicroRNA-203 and its DeltaNp63 Target in Cervical Carcinogenesis: Prospects for Cervical Cancer Screening.* Anticancer Res, 2016. **36**(8): p. 3939–46.
